# Blood Pressure–Lowering by the Antioxidant Resveratrol Is Counterintuitively Mediated by Oxidation of cGMP-Dependent Protein Kinase

**DOI:** 10.1161/CIRCULATIONAHA.118.037398

**Published:** 2019-05-23

**Authors:** Oleksandra Prysyazhna, Kathryn Wolhuter, Christopher Switzer, Celio Santos, Xiaoping Yang, Steven Lynham, Ajay M. Shah, Philip Eaton, Joseph R. Burgoyne

**Affiliations:** 1 King’s College London, School of Cardiovascular Medicine & Sciences, The British Heart Foundation Centre of Excellence, The Rayne Institute, St Thomas’ Hospital, UK (O.P., K.W., C. Switzer., P.E., J.R.B.).; 2 King’s College London, School of Cardiovascular Medicine & Sciences, The British Heart Foundation Centre of Excellence, The James Black Centre, Denmark Hill Campus, UK (C. Santos., A.M.S.).; 3 King’s College London, Proteomics Facility, Centre of Excellence for Mass Spectrometry, The James Black Centre, Denmark Hill Campus, UK (X.Y., S.L.).

**Keywords:** antioxidants, blood pressure, protein kinases, resveratrol, sulfhydryl reagents

## Abstract

**Background:**

The health-promoting and disease-limiting abilities of resveratrol, a natural polyphenol, has led to considerable interest in understanding the mechanisms of its therapeutic actions. The polyphenolic rings of resveratrol enable it to react with and detoxify otherwise injurious oxidants. Whilst the protective actions of resveratrol are commonly ascribed to its antioxidant activity, here we show that this is a misconception.

**Methods:**

The ability of resveratrol to oxidize cGMP-dependent PKG1α (protein kinase 1α) was assessed in isolated rat aortic smooth muscle cells, and the mechanism of action of this polyphenol was characterized using in vitro experiments, mass spectrometry and electron paramagnetic resonance. The blood pressure of wild-type and C42S knock-in mice was assessed using implanted telemetry probes. Mice were made hypertensive by administration of angiotensin II via osmotic mini-pumps and blood pressure monitored during 15 days of feeding with chow diet containing vehicle or resveratrol.

**Results:**

Oxidation of the phenolic rings of resveratrol paradoxically leads to oxidative modification of proteins, explained by formation of a reactive quinone that oxidizes the thiolate side chain of cysteine residues; events that were enhanced in cells under oxidative stress. Consistent with these observations and its ability to induce vasodilation, resveratrol induced oxidative activation of PKG1α and lowered blood pressure in hypertensive wild-type mice, but not C42S PKG1α knock-in mice that are resistant to disulfide activation.

**Conclusions:**

Resveratrol mediates lowering of blood pressure by paradoxically inducing protein oxidation, especially during times of oxidative stress, a mechanism that may be a common feature of antioxidant molecules.

## Abstract 2

Supplemental Digital Content is available in the text.

**Clinical Perspective**

**What Is New?**

- In our study we explain how resveratrol may mediate its numerous beneficial effects, including lowering of blood pressure, by direct thiol oxidation.
- The natural polyphenol resveratrol can counterintuitively induce direct protein oxidation, a process that is enhanced under pro-oxidative conditions associated with disease.
- The oxidation of cGMP-dependent protein kinase 1α (PKG1α) by resveratrol lowers blood pressure in hypertensive mice.

**What Are the Clinical Implications?**

- Our study using a murine model of hypertension demonstrates how blood pressure can be lowered using resveratrol.
- Targeting cysteine 42 on PKG1α may provide a new class of therapeutic that can lower blood pressure in hypertensive patients.
- Identifying additional proteins modified by resveratrol may provide new targets for therapy to treat cardiovascular disease.

Resveratrol is a non-flavonoid polyphenolic compound that was first identified in extracts from the white hellebore *Veratrum grandiflorum*.^1^ After its discovery resveratrol has been characterized as a phytoalexin also found in the skin of several fruits, with the most notable being grapes.^2^ Studies exploring the biological effects of resveratrol have provided compelling evidence for its ability to extend life-span in numerous species, including yeast, worms, flies and mice.^3^ In addition, resveratrol exhibits beneficial effects in diverse animal disease models. This includes the prevention of cardiovascular and neurological diseases, cancer, metabolic syndrome, as well as promoting bone and eye health.^4^ The ability of resveratrol to promote health and prevent disease has predominantly been attributed to its antioxidant activity and to some extent direct target interaction. The most notable direct target of resveratrol being NAD-dependent protein SIRT1 (deacetylase sirtuin-1), which despite being highly publicized, is in fact likely an artefactual result of an in vitro activity assay used in the original study.^4–7^ Despite this setback genuine direct targets of resveratrol have been subsequently identified and include cAMP phosphodiesterases and tyrosyl transfer-RNA synthetase.^8,9^ Although these targets of resveratrol are known, the biological mechanisms that mediate this polyphenol’s protective signaling are yet to be fully elucidated. Here we provide a novel thiol-dependent mechanism that mediates, at least in part, the beneficial actions of resveratrol. This mechanism involves the oxidation of thiols that includes a new biological target, which mediates the vasodilatory properties of this polyphenol, namely PKG1α (protein kinase 1α). This ability of resveratrol to modify cysteine thiols was catalyzed by superoxide, the formation of which is often causatively associated with disease.^10–13^ Therefore, this thiol-mediated activity of resveratrol is likely to be restricted to the pro-oxidative environment of diseased tissues, thus mediating beneficial signaling at the site of injury. Consequently, this study provides novel insight as to how resveratrol promotes health and limits disease, as well as highlighting the ability of antioxidants to mediate protective signaling by counterintuitively inducing thiol oxidation.

## Methods

An extended methods section is available in the online-only Data Supplement. The data, analytical methods, and study materials will be made available to other researchers for purposes of reproducing the results or replicating the procedure and are available by contacting the corresponding author.

### Measuring PKG1α Disulfide Dimerization

1.15 μg/μl of recombinant PKG1α (Merck Millipore) was reduced for 30 minutes by addition of 2 mmol/L dithiothreitol. After reduction PKG1α was diluted to 24.5 ng/μl in 100 mmol/L Tris-HCl pH 7.4. Diluted PKG1α was then exposed to 100 μmol/L resveratrol (Sigma) or DMSO. After 30 minutes incubation at room temperature reactions were terminated by the addition of sample buffer (50 mmol/L Tris-HCl buffer pH 6.8, 2% SDS, 10% glycerol, 0.005% bromophenol blue) containing 100 mmol/L maleimide to prevent further PKG1α oxidation. Samples were then resolved on SDS-PAGE gels and immunoblotted for PKG1α (Enzo Life Sciences).

### Cell Culture

Rat aortic smooth muscle cells (SMC) were cultured in Dulbecco’s Modified Eagle’s medium supplemented with 1% Penicillin-Streptomycin and 10% fetal bovine serum. Human primary vascular SMC were cultured in medium 199 supplemented with 1% penicillin-streptomycin and 20% fetal bovine serum. SMC were maintained at 37^o^C in a 20%O_2_/5%CO_2_ incubator and grown on 12-well culture plates until 90% confluent (approximately 0.3 x 10^6^) before experimental treatment. In some experiments, cells were maintained at 5%O_2_/5%CO_2_. Cultured SMC were treated with freshly prepared resveratrol or resveratrol pre-exposed to 25 minutes of UV irradiation (Blak-Ray B-100AP high intensity UV lamp). In some experiments SMC were treated with 10 μmol/L menadione sodium bisulfite (Sigma) alone or with 100 μmol/L resveratrol. In all experiments SMC were lysed 25 minutes after treatment in sample buffer containing 100 mmol/L maleimide. To assess PKG1α intramolecular disulfide formation, SMC were pretreated with resveratrol for 15 minutes then a further 10 minutes with 200 μmol/L 1-nitrosocyclohexyl acetate.

### Electron Paramagnetic Resonance

Resveratrol (20 mmol/L) or equivalent volume of DMSO in PBS was supplemented with 17 mmol/L 2-methyl-2-nitrosopropane in the presence of absence of tyrosinase. The formation of 2-methyl-2-nitrosopropane adducts was analyzed using electron paramagnetic resonance (Magnettech Miniscope MS2000 spectrometer) after 160 minutes incubation at room temperature.

### Animals

All procedures were performed in accordance with the Home Office Guidance on the Operation of the Animals (Scientific Procedures) Act 1986 in the United Kingdom. Experiments were approved by the King’s College London Animal Welfare and Ethical Review Body. Mice constitutively expressing PKGIα Cys42Ser were generated on a pure C57BL/6 background by TaconicArtemis (Köln, Germany) as previously described.^14^ Wild-type or littermate C42S PKG1α knock-in (KI) male mice were used at 14–15 weeks of age.

### Isolated Mesenteric Vessel Preparation

Mesenteric arteries from wild-type or C42S PKG1α KI mice were isolated, cleaned of adipose and connective tissue then mounted in a Danish Myo Technology tension myograph system. Mounted vessels submerged in Krebs solution were stretched to their optimal pre-tension using the tension myograph normalization module at 37°C containing 95% O_2_:5% CO_2_. The vasotone of each vessel to increasing concentrations of resveratrol was measured after wake-up with KCl (60 mmol/L) and after constriction to U46619 (0.1 μmol/L).

### Angiotensin II–Induced Hypertension and Resveratrol Feeding

Mice were randomized and data analysis was blinded to the operator. No animals were excluded after the randomization. Groups were age and weight matched. After baseline recordings (4 days) mice were again anesthetized (2% isoflurane in 0.5 L of oxygen/minute) and subjected to subcutaneous implantation of osmotic minipumps (model 1002; Alzet) for delivery of angiotensin II (Sigma) at an infusion rate of 1.1 mg/kg/day. Perioperative analgesia (buprenorphine, 0.1 mg per kg of body weight; Abbot Laboratories) was provided, and the wellbeing of mice monitored daily. Resveratrol was delivered orally (~320mg/kg) and to mitigate stress or risk of dislodging the telemetric probe catheter, it was provided suspended in water and set in gelatin flavored with sodium saccharin and mixed with chow. Food consumption was monitored daily, which on average did not vary between genotypes. At the end of the feeding study, mice were euthanized and mesenteries isolated for analysis.

### Statistical Analysis

Results are presented as mean±SE. Differences between 2 groups were assessed using a 2-tailed unpaired Student *t* test or for multiple comparisons an ANOVA followed by Tukey post hoc analysis. All datasets had a normal distribution, as confirmed using the Shapiro-Wilk normality test. Results were considered significant with *P* values ≤ 0.05.

## Results

**Figure 1.**
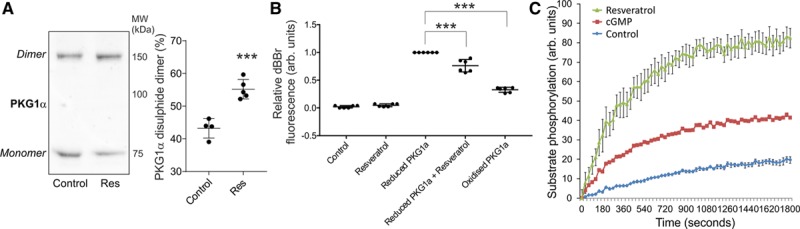
**Resveratrol induces direct PKG1α oxidation and activation. A**, Recombinant PKG1α undergoes disulfide dimerization when exposed to resveratrol (n=4–5). **B**, Resveratrol prevents modification of vicinal thiols on PKG1α by dibromobimane (dBBr), which emulated air oxidation (n=6). **C**, Resveratrol induces cGMP-independent stimulation of PKG1α activity consistent with oxidative activation (n=4). ***P<0.005. All data are presented as mean±SE. and analyzed using unpaired Student *t* test (A) or 1-way ANOVA followed by Tukey.

Resveratrol alone induced a significant increase in recombinant PKG1α disulfide formation, observed on immunoblots and by a loss in reduced vicinal thiols detected by a decrease in dibromobimane fluorescence (Figure 1A and 1B). Consistent with resveratrol inducing PKG1α oxidation, the catalytic activity of the kinase was also enhanced independently of cGMP (Figure 1C).^15^

**Figure 2.**
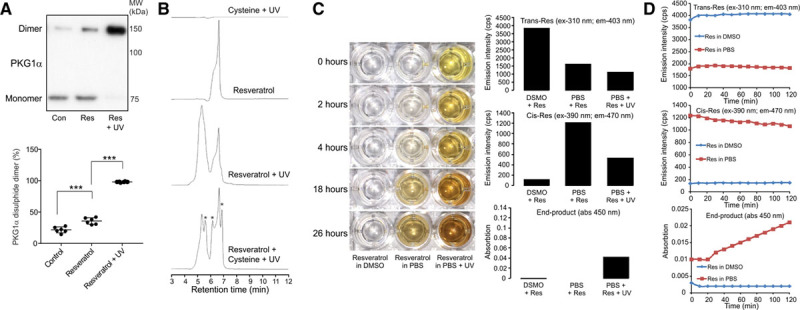
**Exposure of resveratrol to UV irradiation enhances its ability to mediate thiol oxidation.** **A**, PKG1α oxidation in SMC is significantly enhanced when resveratrol is pre-exposed to UV irradiation (n=6). **B**, UV irradiation of resveratrol converts it from the trans to the cis form as previously described. However, in the presence of cysteine new products are formed (*) consistent with cysteine adducted by resveratrol. **C**, When placed into aqueous solution resveratrol is slowly converted into a colored end-product that is catalyzed by initial UV irradiation. The initial detection of trans, cis and end-product forms of resveratrol is shown in each graph. **D**, The addition of resveratrol to aqueous solution immediately induces partial isomerization of the trans to cis form. This cis form is then time-dependently lost with formation of an end-product. ***P<0.005. All data are presented as mean±SE. and analyzed using 1-way ANOVA followed by Tukey (**A**).

The ability of resveratrol to induce PKG1α oxidation was substantiated in primary cultured rat aortic SMC and human vascular SMC exposed to this polyphenol. Treatment of SMC with resveratrol induced PKG1α oxidation, which was significantly enhanced when resveratrol was pre-exposed to ultraviolet (UV) light (Figure 2A and Figure IA through IC in the online-only Data Supplement). The oxidation of PKG1α by resveratrol induced a Cys42-dependent intermolecular disulfide, but not an intramolecular bond between Cys117 and Cys195 (Figure ID in the online-only Data Supplement). In addition, resveratrol did not prevent the intramolecular disulfide between Cys117 and Cys195 that was induced by the HNO donor,^16^ 1-nitrosocyclohexyl acetate. Therefore, the oxidation of PKG1 by resveratrol is likely to be selective for cysteine 42. As well as inducing PKG1α oxidation, resveratrol also caused disulfide dimerization of the regulatory I-α subunit of cAMP-dependent protein kinase (PKARI; Figure IE and IF in the online-only Data Supplement).^17^ Therefore, protein oxidation is likely a generic mechanism for mediating resveratrol-induced signaling. The ability of resveratrol to induce protein oxidation through a direct thiol adduction mechanism was assessed using reverse phase liquid chromatography. The modification of cysteine by resveratrol was observed after UV light exposure, as indicated by detection of additional, nascent products (Figure 2B). To provide further insight to the mechanism by which resveratrol adducts thiols, the presence of it trans or cis isomers was assessed based on their differential fluorescence properties, as determined using commercial standards (Figure IIA in the online-only Data Supplement). The addition of resveratrol to an aqueous solution led to its spontaneous partial isomerization from the trans to the cis form (Figure 2C and 2D and Figure IIA and IIB in the online-only Data Supplement). The cis isomer generated after addition of aqueous solution, was lost over time and correlated with increased end-product formation measured at 450 nm. This is consistent with the spontaneous conversion of cis-resveratrol to the end-product form, with this process catalyzed by UV light. In addition, the spontaneous isomerization of resveratrol and slow accumulation of end-product upon addition of aqueous PBS did not occur in DMSO. The colored end-product formed in aqueous solution was consistent with resveratrol dimerization.^18,19^ This was supported by its similarity in color and absorbance to viniferin, with the end-product also likely comprising of other various oligomers (Figure IIC in the online-only Data Supplement). These changes in the chemical composition of the polyphenol upon addition to aqueous solution was substantiated using additional commercial sources of trans-resveratrol (Figure III in the online-only Data Supplement). For each commercial source of trans-resveratrol tested, partial isomerization to the cis form occurred spontaneously upon addition to aqueous PBS. This cis isomer was then lost with time, which correlated with increased formation of an end-product that absorbed at 450 nm.

**Figure 3.**
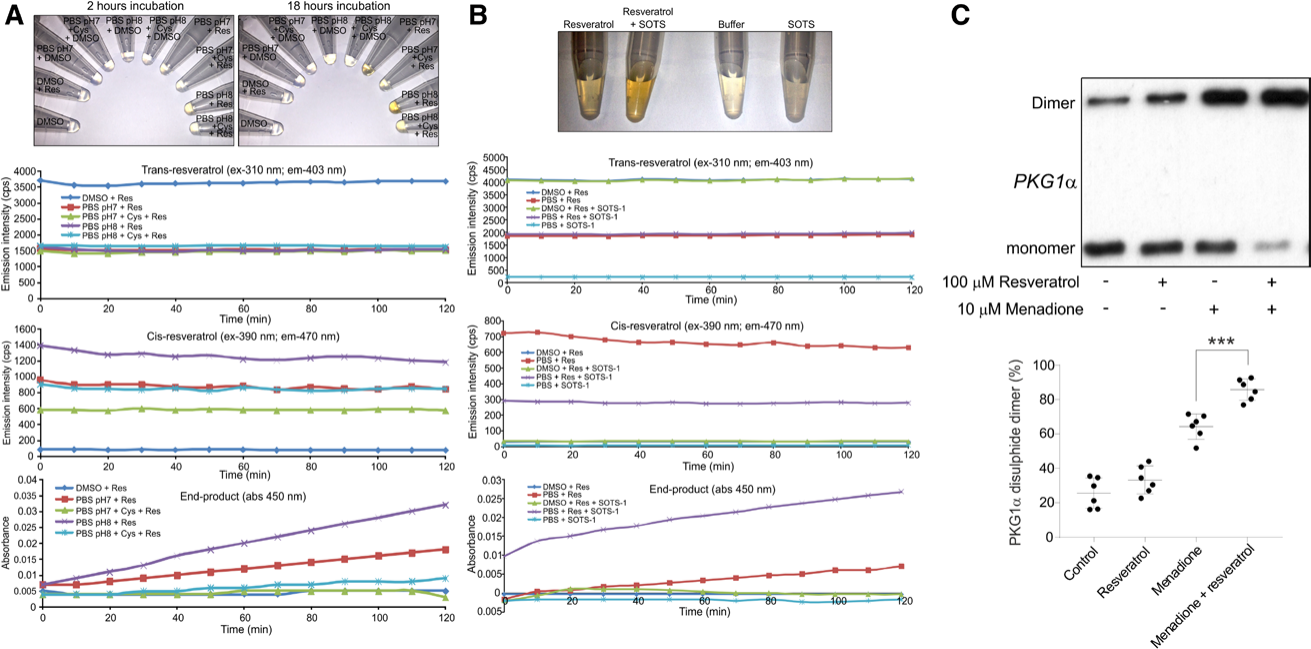
**Superoxide catalyzes resveratrol end-product formation and its ability to oxidize PKG1α. A**, Cysteine attenuates the conversion of resveratrol to its end-product consistent with a thiol-reactive intermediate. **B**, The addition of the superoxide donor SOTS-1 to resveratrol rapidly enhances end-product formation. **C**, The oxidation of PKG1α mediated by menadione is substantially enhanced by addition of resveratrol (n=6). ***P<0.005. All data are presented as mean±SE. and analyzed using 2-way ANOVA followed by Sidak post hoc test (**C**).

Upon addition to aqueous solution, an intermediate form of resveratrol with thiol reactivity was likely generated, as end-product formation was inhibited by co-incubation with L-cysteine (Figure 3A). Furthermore, resveratrol end-product formation and loss of the cis isoform was catalyzed by superoxide generated by SOTS-1 (Figure 3B). Therefore, it is conceivable that oxidation of resveratrol may catalyze formation of a thiol-reactive intermediate, which in isolation resolves to form the end-product. The ability of superoxide to enhance formation of a thiol reactive resveratrol intermediate was further supported by experiments using menadione. Although menadione can induce PKG1α oxidation in SMC alone, through elevated oxidant formation, this was substantially enhanced in the presence of resveratrol (Figure 3C).

**Figure 4.**
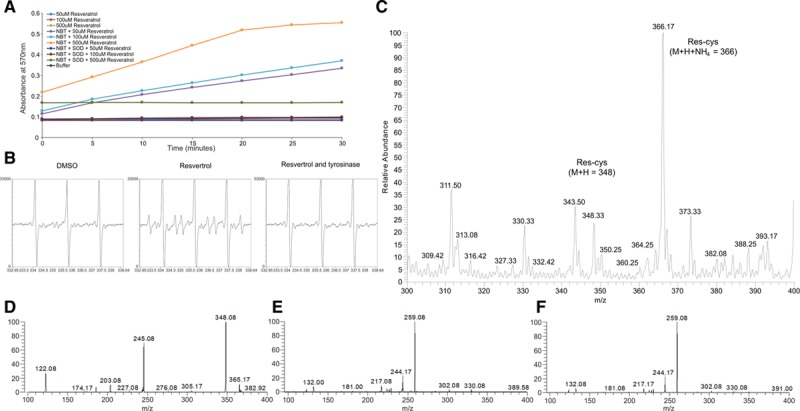
**Resveratrol is converted into a semiquinone when placed into aqueous solution and can directly modify cysteine. A**, The conversion of reduced nitroblue tetrazolium (NBT) to its formazan form is enhanced in a time and concentration-dependent manner by resveratrol, which is attenuated by superoxide dismutase (SOD), thus consistent with superoxide formation. **B**, In aqueous solution resveratrol is converted to a semiquinone radical that modifies 2-methyl-2-nitrosopropane (MNP), which is absent in samples supplemented with tyrosinase. **C**, Product spectra from positive-ion electrospray mass spectrometry analysis of cysteine adducts after incubation with resveratrol. Here products with a mass consistent with cysteine covalent bound to resveratrol alone and with an ammonium ion adduct were detected. **D**, Fragmentation of product ion m/z 366 from **C**. **E**, Fragmentation of product m/z 348 from **D**. **F**, Fragmentation of product ion m/z 348 from **C**.

The ability of resveratrol to mediate protein oxidation and undergo dimerization is consistent with redox cycling between a semiquinone (phenoxyl radical) and quinone form, which is also attributed to its antioxidant activity.^18–22^ This is also consistent with increased end-product formation when resveratrol is deprotonated to the phenolate anion form under basic pH, which will catalyze semiquinone formation (Figure IV in the online-only Data Supplement). The generation of superoxide consistent with redox cycling of resveratrol between a semiquinone and quinone form, was detected by the reduction of nitro-blue tetrazolium, which was attenuated by superoxide dismutase (Figure 4A). Spontaneous formation of a resveratrol semiquinone upon addition of aqueous solution, was further substantiated using electron paramagnetic resonance. Incubation of resveratrol with 2-methyl-2-nitrosopropane generated a composite electron paramagnetic resonance spectrum whose main components comprised a 3-line light-induced (a^N^ = 16.5 G) and a 6-line spectrum (a^N^ = 13.5 G; aH = 16.9 G) characteristic of a carbon centered adduct (Figure 4B). As tyrosinase catalyzes quinone formation by 2 electron oxidation this will limit semiquinone formation. Therefore, as the novel 2-methyl-2-nitrosopropane adduct was absent in samples when also supplemented with tyrosinase, this substantiates formation of a resveratrol semiquinone. The redox cycling of resveratrol to a quinone form may underlie its capacity to modify cysteine, as it would confer an ability to adduct to thiols through a Michael addition reaction across its α,β-unsaturated carbonyl. This was investigated by analyzing the formation of cysteine resveratrol adducts using mass spectrometry. Here the incubation of cysteine with resveratrol led to formation of products consistent with thiol covalently bound to the polyphenol, which can be observed in the mass spectrum with or without the addition of an ammonium ion adduct (Figure 4C). The formation of resveratrol covalently bound to cysteine was further substantiated, as additional products were absent in samples containing cysteine or resveratrol alone (Figure VA and VB in the online-only Data Supplement). In addition, the formation of an ammonium adduct of cysteine bound resveratrol (m/z 348) was confirmed by tandem mass spectrometry analysis. Fragmentation of the product at m/z 366 generated a dominant ion at m/z 348 (Figure 4D), which after further fragmentation matched the profile for the m/z 348 ion for cysteine bound resveratrol (Figure 4E and 4F). Here the addition of resveratrol also induced a relative increase in the abundance of cystine, thus further supporting the ability of this polyphenol to directly induce disulfide formation (Figure VC in the online-only Data Supplement). This capacity of resveratrol to catalyze a disulfide between 2 cysteine molecules is consistent with a direct reaction of the polyphenol with PKG1α Cys42 that induces intermolecular disulfide formation. As resveratrol also directly catalyzed the S-glutathiolation of recombinant glyceraldehyde 3-phosphate dehydrogenase, this further corroborates the ability of this polyphenol to generically induce disulfide formation (Figure VIA in the online-only Data Supplement).

**Figure 5.**
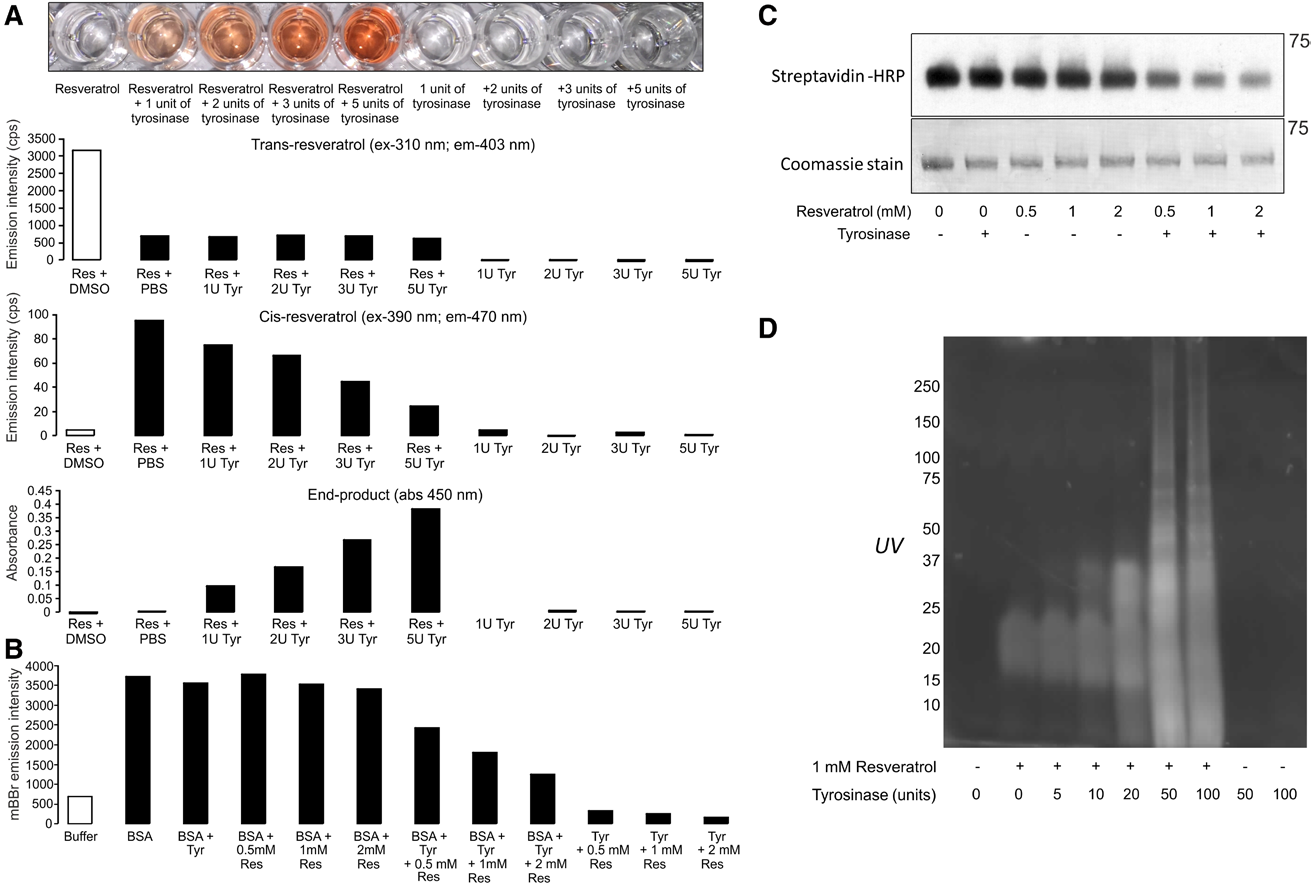
**A resveratrol semiquinone intermediate modifies protein thiols. A**, Addition of tyrosinase (Tyr) enhances end-product formation visualized after 5 minutes incubation. **B**, Resveratrol modifies reduced thiols on bovine serum albumin in a concentration-dependent manner that is enhanced by tyrosinase, assessed by a loss in conjugated monobromobimane fluorescence. **C**, The loss of free thiols on BSA after exposure to resveratrol was substantially enhanced by tyrosinase, as observed by a decrease in biotin-maleimide conjugation. **D**, Modification of proteins by resveratrol within cell lysate is substantially enhanced by tyrosinase, observed after SDS-PAGE separation followed by exposure of proteins to UV irradiation.

The ability of resveratrol to modify cysteine through a reactive intermediate was further investigated, by selectively catalyzing quinone formation using the phenol oxidase enzyme tyrosinase. The 2-electron oxidation of resveratrol by tyrosinase led to rapid end-product formation and loss of the cis form, consistent with enhanced dimerization (Figure 5A). In addition, the quinone form of resveratrol generated by tyrosinase was highly reactive and led to loss of free thiols in BSA, detected by a decrease in either monobromobimane fluorescence or biotin-maleimide labeling (Figure 5B and 5C). The ability of a resveratrol quinone to modify proteins was further substantiated in cell lysates by detection of the polyphenol conjugated to proteins on SDS-PAGE gels irradiated with UV (Figure VIB in the online-only Data Supplement). This modification of proteins by resveratrol was dramatically enhanced by addition of tyrosinase, consistent with enhanced thiol reactivity of the quinone form (Figure 5D).

**Figure 6.**
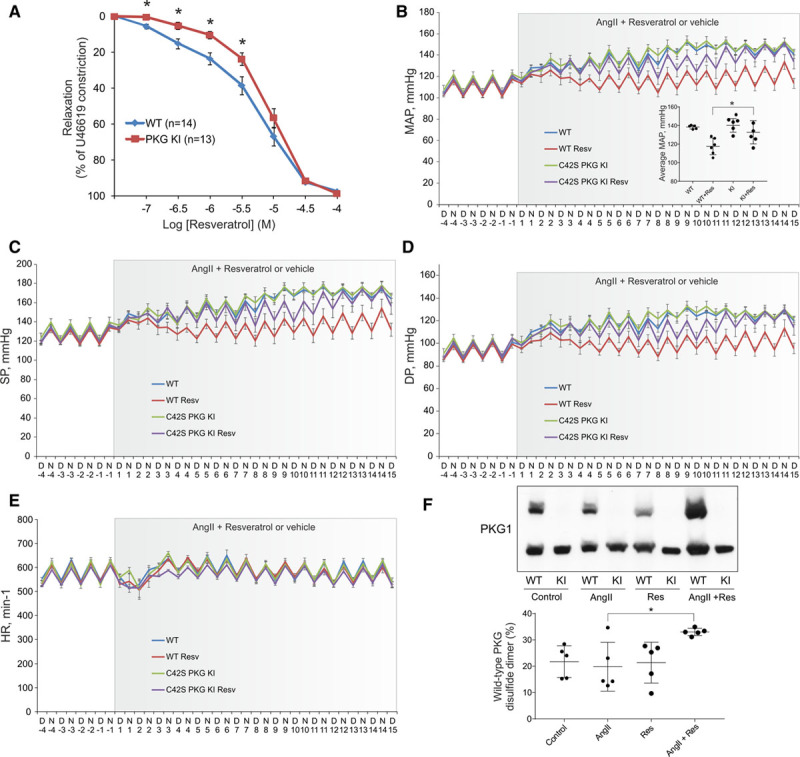
**PKG1α oxidation mediates the vasodilatory action of resveratrol.** **A**, Resveratrol induced vasodilation of constricted mesenteric vessels that was significantly impaired in those isolated from C42S KI mice (n=5–6). **B**, Feeding hypertensive mice with resveratrol significantly lowers mean arterial pressure (MAP) in wild-type (WT) but not C42S PKG1α KI mice (n=5–6). **C** and **D**, Feeding of resveratrol lowers systolic (SP) and diastolic pressure (DP) to a greater extent in hypertensive wild-type compared with C42S PKG1α KI mice (n=5–6). **E**, Feeding of resveratrol does not impact on heart rate (HR; n=5–6). **F**, Feeding resveratrol increases PKG1α oxidation in angiotensin II (AngII) hypersensitive wild-type mice (n=5–6). *P<0.05. All data are presented as mean±SE and analyzed using 2-way ANOVA followed by Sidak post hoc test.

The ability of resveratrol to induce PKG1α oxidation mediated to some extent its vasodilatory action in isolated constricted mesenteric vessels (Figure 6A). Here resveratrol-induced vasorelaxation was impaired in vessels from C42S PKG1α KI mice when compared with littermate wild-types. Consistent with direct oxidative activation of vascular smooth muscle cell PKG1α, the relaxation to resveratrol in L-NAME-treated or endothelium denuded vessels was comparable to control tissue (Figure VIIA in the online-only Data Supplement). The ability of resveratrol to mediate vasodilation and thus lower blood pressure was further assessed in angiotensin II–induced hypertensive mice monitored telemetrically. Here oral feeding of resveratrol significantly attenuated angiotensin II–induced hypertension in wild-type but not C42S PKG1α KI mice (Figure 6B through 6D). However, feeding of resveratrol did not impact on heart rate or mouse activity (Figure 6E and Figure VIIB in the online-only Data Supplement). In mice fed resveratrol, plasma serum albumin was modified by this polyphenol, which was detected by mass spectrometry (Figure VIIC and VIID in the online-only Data Supplement). This finding corroborates the capacity of resveratrol to directly modify protein thiols in mice that consume it. Resveratrol-induced protein oxidation likely explains its ability to lower blood pressure in hypertensive mice, with enhanced PKG1α disulfide dimerization being a principal causative mechanism. This was substantiated by disulfide dimerization of PKG1α in the mesenteric vessels of resveratrol fed mice, which was significantly enhanced in wild-type animals also administered angiotensin II (Figure 6F). As enhanced PKG1α oxidation mediated by resveratrol was only observed in wild-type hypertensive mice, it is likely this process is driven by oxidation of the polyphenol, mediated by angiotensin II–induced superoxide formation.^23^ Indeed, as resveratrol was found to be largely stable in the food preparation, this further supports the likelihood of polyphenol oxidation in vivo, which in turn will induce PKG1α disulfide dimerization (Figure VIIE in the online-only Data Supplement).

## Discussion

The medicinal benefits of resveratrol have been extensively characterized in animal models of health and disease, however it has proven difficult to translate these findings to the treatment of human disease. Although there have been some promising outcomes in the treatment of Alzheimer disease, which suggest it can modulate neuro-inflammation and induce adaptive immunity.^24^ Despite resveratrol having so far failed to translate to a clinical use, which is largely attributed to its poor bioavailability, the beneficial effects in animal models, including extension in life-span, warrants a greater understanding of its biological activity.^25,26^ Certainly, improved insight into the mechanistic basis for the actions of resveratrol, could provide new therapeutic strategies with improved potency in humans.

In this study, we examined the potential for resveratrol to mediate at least in part its beneficial effects through oxidative signaling. The rational being phenolic antioxidants are innately, and often overlooked, pro-oxidants due their ability to redox cycle.^27^ Indeed, in the case of resveratrol a pro-oxidative activity has already been attributed.^28,29^ Resveratrol alone induces oxidative DNA damage that is enhanced by transition metal ions including copper.^30^ Indeed, this ability of resveratrol to induce oxidative DNA damage has been implicated as a possible mechanism for its anticancer activity.^31^ The polyphenols pro-oxidative activity is likely through reactive species formation upon electron transfer from resveratrol to oxygen, leading to conversion of its phenolic hydroquinone to a semiquinone.

Here we found resveratrol alone was enough to induce oxidation and subsequent activation of recombinant PKG1α. This was consistent with auto-oxidation of resveratrol in solution, thus generating a thiol-reactive intermediate or reactive oxygen species that would catalyze disulfide formation. Consistent with the auto-oxidation of resveratrol we observed a slow chemical shift upon its addition to aqueous solution, characterized by increased coloration and absorbance at 450 nm. This chemical change was likely explained by formation of the resveratrol dimer viniferin through semiquinone attack of a second resveratrol molecule or free radical coupling.^18,19^ This observation resonates with a recent study where resveratrol dimers and tetramers were synthesized using a resveratrol radical.^32^ As with this study it is likely that the end-products we observed also compromises a mixture of resveratrol derivatives because of divergent cyclisation pathways. The formation of superoxide upon addition of resveratrol to aqueous solution was again consistent with a semiquinone intermediate that redox cycles between its hydroquinone form. Certainly, a semiquinone is also a likely intermediate in the reaction of resveratrol with superoxide, which is considered crucial to its well-described antioxidant activity. This has been described through two potential mechanisms, one consisting of hydrogen-atom transfer and the other through a single electron transfer reaction.^20–22^ Studies assessing the antioxidant activity of resveratrol using density functional theory, determined that because of resonance the 4’-OH group has greater reactivity than those on the opposing ring in the 3- or 5- positions.^33^ In addition, the antioxidant activity of resveratrol is attributed to formation of a semiquinone in which the unpaired electron is mainly distributed on the O-atom at the para position. The importance of the 4’-OH group in mediating the antioxidant activity of resveratrol is further supported by quantum-chemical calculations, in which this group was shown to be a strong proton donor and capable of forming a semiquinone.^34^

**Figure 7.**
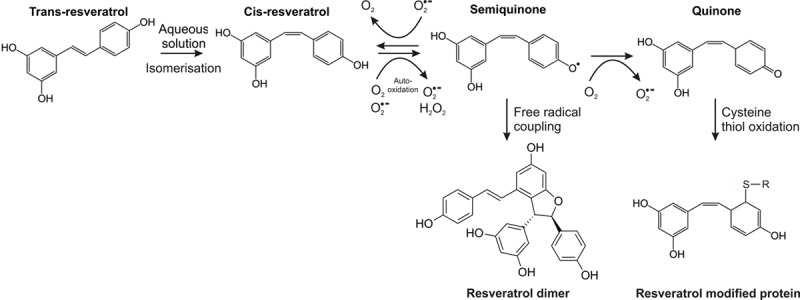
**Overview of thiol-dependent mechanism of resveratrol action.** The addition of trans-resveratrol to aqueous solution induces spontaneous isomerization to the cis form. Once formed cis-resveratrol auto-oxidizes to a semiquinone that is catalyzed by superoxide. The unstable semiquinone intermediate transitions to a quinone form or dimerizes through a free radical coupling reaction. A resveratrol quinone generated through polyphenol oxidation has the propensity to modify protein cysteine thiols through a Michael addition reaction.

The ability of UV exposure to enhance resveratrol-mediated cysteine thiol oxidation is likely through increased formation of its quinone form. Indeed, this is consistent with enhanced end-product formation through attack of a second resveratrol molecule by a semiquinone intermediate. In addition, the spontaneous formation of a semiquinone intermediate of resveratrol upon addition to aqueous solution seems to require isomerization of the trans to cis form. This is evident from a time-dependent loss of the cis isomer with accumulation of its end-product. It is conceivable that this could be explained by enhanced sensitivity of the cis form to auto-oxidation. Indeed, this could also explain why UV light enhances the thiol reactivity of resveratrol, by increasing isomerization to the cis form, which is then subsequently converted to a quinone. The oxidation of resveratrol is also enhanced by superoxide formation as the donor SOTS-1 rapidly increased end-product formation. This is consistent with its hydrogen-atom transfer or single electron transfer reaction.^20–22^ These findings suggest that under biological conditions where superoxide formation is elevated, as occurs in many prevalent diseases, resveratrol will be converted to a thiol-reactive quinone form. Certainly, this would be predicted as the catalytic conversion of resveratrol to a quinone form using tyrosinase, leads to enhanced protein modification. The modification of proteins by resveratrol under pro-oxidizing conditions may underlie its ability to mediate protective signaling. Indeed, this ability of resveratrol to modify protein thiols is consistent with the adduction of glutathione within the human liver microsome.^35^ This modification of glutathione by resveratrol, detected using mass spectrometry, was consistent with a thiol reactive quinone form. The chemistry required for modification of glutathione by resveratrol will not differ from that of a protein thiol. Therefore, it is perhaps unsurprising that modification of proteins by resveratrol was observed and was enhanced by tyrosinase. Here we were able to detect cysteine modified by resveratrol using mass spectrometry, and proteins modified by resveratrol using UV irradiation, which provides a novel strategy that can be utilized to further investigate this new mode of signaling. The modification of proteins by resveratrol may in part underlie its biological effects, which will be potentiated under pro-oxidizing conditions associated with prevalent diseases, because of enhanced redox cycling and quinone formation. Therefore, the numerous beneficial actions of resveratrol attributed to its antioxidant activity is likely a misconception, as this is contradicted by the formation of an oxidized thiol reactive intermediate that promotes oxidative signaling. As the thiol reactivity of a resveratrol quinone will be constrained by the structural and electrochemical properties surrounding susceptible cysteine residues, this will limit the targets that undergo this modification. Therefore, in diseased tissue it is likely resveratrol converts a non-selective oxidant into a discriminatory form that mediates beneficial oxidative signaling to promote health. Furthermore, the well-documented ability of resveratrol to prevent disease by lowering oxidative stress is also likely a misconception. This is because oxidative stress is normally based on the measure of a select oxidant (eg, superoxide) or post-translational modification (eg, protein carbonylation), whereas protein modification by quinones is not normally assessed. This counterintuitive role of resveratrol means that despite its ability to lower common markers of oxidative stress, it is likely protein oxidation by this polyphenol is enhanced under disease conditions. The proposed mechanism of thiol adduction by resveratrol is shown in Figure 7.

It is likely that resveratrol modifies multiple targets in addition to PKG1α, with the rational possibility that these may also mediate its beneficial effects. Consistent with this idea, numerous proteins were modified by resveratrol in a tyrosinase-catalyzed reaction as observed under UV irradiation. In addition, PKARI also underwent disulfide dimerization in SMC treated with resveratrol, identifying this kinase as another novel target of this polyphenol. Based on these findings, it is also conceivable that some of the known processes regulated by resveratrol could be attributed to its ability to modify protein cysteine thiols. This may include its ability to regulate NAD(P)H oxidase activity, or eNOS (endothelial nitric oxide synthase) phosphorylation, an event that improves endothelial function in type 2 diabetic mice.^36^ Also, protein thiol modification could contribute to prevention of stretch-induced high-mobility group box 1 release by resveratrol, which protects against lung endothelial barrier dysfunction.^37^ As this protective mechanism is dependent on Nrf2 (nuclear factor erythroid 2-related factor 2), it is conceivable that resveratrol could modify cysteines on KEAP1 (kelch-like ECH-associated protein 1), thus enabling the stabilization of Nrf2. This may be anticipated, as KEAP1 is well known to generically react with low molecular weight electrophiles, such as sulforaphane.^38^ This mechanism may also underlie the ability of resveratrol to induce expression of antioxidant genes, through thiol-dependent activation of Nrf2.

The disulfide dimerization of PKG1α may be induced by resveratrol through a polyphenol conjugated thiol intermediate. Alternatively, disulfide formation may be mediated by 1 electron oxidation of cysteine 42 of PKG1α to a thiyl radical by a semiquinone resveratrol intermediate. The formation of a thiyl radical then leads to generation of a disulfide through reaction with an adjacent reduced thiol, and the donation of an electron to oxygen.^39^ However, superoxide generated upon resveratrol auto-oxidation could also mediate disulfide formation through its conversion to H_2_O_2_, by spontaneous dismutation or catalyzed by cellular superoxide dismutase.^40^ This is consistent with previous studies showing H_2_O_2_ can induce PKG1α disulfide dimerization and activation.^15,41^ The contribution of each process in resveratrol-mediated PKG1α disulfide dimerization is difficult to assess, as it is not possible to isolate and identify the intermediates involved. Despite this limitation we have identified PKG1α as a bona fide novel target of resveratrol, in which the polyphenol promotes activation of the kinase by inducing disulfide dimerization. This mechanism of oxidative PKG1α activation mediates in part the biological activity of resveratrol. In constricted mesenteries, the ability of resveratrol to mediate vasorelaxation was partially impaired in those expressing a redox-dead C42S form of PKG1α. This ability of resveratrol to relax mesenteric vessels was also found to be endothelium-independent, thus further supporting the potential role of vascular SMC PKG1α oxidation in mediating the biological actions of this polyphenol. Although only a minor difference in relaxation between wild-type or C42S KI vessels was observed, these were healthy vessels and therefore resveratrol redox cycling may be limited. It is therefore conceivable that in diseased vessels where oxidant formation is elevated that the relaxation to resveratrol would be potentiated by its ability to further enhance PKG1α oxidation. This is consistent with experiments using the oxidant generator menadione, where its ability to oxidize PKG1α was substantially enhanced in cells co-treated with resveratrol. Based on these findings, we predicted that resveratrol would lower blood pressure in angiotensin II hypertensive mice by enhancing the oxidation of PKG1α. Here the rationale was superoxide formation associated with the pathological effects of angiotensin II would potentiate redox cycling of resveratrol,^23^ which would then couple to enhanced PKG1α oxidation to lower blood pressure. Consistent with previous studies, we found that feeding resveratrol effectively lowered blood pressure in hypertensive wild-type mice.^42,43^ Importantly and in accordance with our hypothesis, we found that this lowering of blood pressure, mediated by resveratrol, was absent in C42S PKG1α KI mice. The role of PKG1α disulfide dimerization in mediating the blood pressure lowering action of resveratrol was further substantiated in mesenteric vessels. An increase in PKG1α oxidation mediated by resveratrol was observed only in wild-type hypertensive mouse mesenteric vessels, therefore it is likely this process is driven by oxidation of the polyphenol, mediated by angiotensin II–induced superoxide formation.^23^ This ability of resveratrol to lower blood pressure may also be mediated by oxidative modulation of other proteins in addition to PKG1α. This includes cysteine thiols on PKARI, which were also found to undergo polyphenol-induced oxidation.^44^

A limitation of this study is the lack of patient data, which is important to establish that animal findings can be translated to humans. However, as resveratrol induced PKG1α oxidation in human vascular SMC, it is anticipated that our findings will be translatable to the treatment of human hypertension. This is also consistent with H_2_O_2_-dependent dilation of human coronary arterioles being mediated by the disulfide dimerization of PKG1α.^45^ Therefore, based on our findings we predict that under disease conditions with altered reactive oxygen species bioavailability or pH, the ability of resveratrol to induce protein oxidation may be enhanced, thus potentiating beneficial signaling. It is also important to bear in mind that as well as protein oxidation, resveratrol through a similar mechanism may also induce DNA damage,^28,30^ which could have a deleterious effect that should be carefully assessed before conducting human studies. Together, our findings provide a novel mechanism that underlies the ability of resveratrol to lower blood pressure, which is through oxidation of C42 on PKG1α. This observation is also consistent with our previous work where we identified a thiol-reactive drug termed G1, which likewise lowers blood pressure through oxidation of C42 on PKG1α.^46^ Therefore, based on our observations and the numerous health benefits of resveratrol, protein cysteine thiols may represent promising targets for future drug development.

In summary, here we describe a new paradigm in antioxidant signaling as resveratrol can paradoxically mediate its biological actions through protein oxidation, which is because of its ability to redox cycle. A new target of this novel mode of signaling is C42 on PKG1α, which when oxidized by resveratrol lowers blood pressure in hypertensive animals.

## Sources of Funding

This study was supported by the British Heart Foundation (sponsor reference FS/14/1/30551), the Medical Research Council, and the European Research Council.

## Disclosures

None.

## Supplementary Material
