# Supplementary material for: Blood Pressure–Lowering by the Antioxidant Resveratrol Is Counterintuitively Mediated by Oxidation of cGMP-Dependent Protein Kinase
Source: Circulation. 2019 May 23;140(2):126–37. doi: 10.1161/CIRCULATIONAHA.118.037398 (PMC6635045; doi:10.1161/CIRCULATIONAHA.118.037398)
Supplement: Supplementary file 1 [file cir-140-126-s001.pdf]

SUPPLEMENTAL MATERIAL

Supplementary methods

Dibromobimane assay

1.1 μg/μl of recombinant PKG1α was reduced by treating with 5 mM tris(2-carboxyethyl)phosphine (TCEP) or air oxidised for 20 minutes at room temperature. Reduced and oxidised PKG1α was diluted to 75 ng/μl in 100 mM Tris-HCl pH 7.4 in the presence or absence of 200 μM resveratrol, and then incubated for 30 minutes at room temperature. To each sample 100 μM di-bromobimane (ThermoFisher Scientific) was then added before a further 60-minute incubation. After incubation the fluorescence of di-bromobimane (λex 393 nm; λem 477 nm) in each sample was assessed using a microplate reader (SpectraMax GeminiXS, Molecular Devices).

PKG1α activity assay

700 ng/μl Recombinant PKG1α was reduced with 4 mM dithiothreitol (DTT) for 20 minutes at room temperature. After reduction PKG1α was diluted to 26 ng/μl in Omnia assay buffer (ThermoFisher Scientific) that in some cases was supplemented with 20 μM cGMP, 100 μM freshly prepared resveratrol or DMSO. The activity of PKG1α was then assessed every 30 seconds over 30 minutes at room temperature by measuring substrate fluorescence (λex 360 nm, λem 485 nm) using a microplate reader

In vitro experiments

60 mM *trans*-resveratrol (Sigma) or *cis*-resveratrol (Cayman Chemical) solublised in DSMO was diluted to 20 mM in phosphate buffered saline (PBS) or DMSO then immediately analysed using a microplate plate reader for its fluorescence (SpectraMax GeminiXS, Molecular Devices) or absorbance at 450 nm (BioTek EL808 plate reader). In some experiments, resveratrol was either exposed to UV irradiation or assessed at varying pH. To examine the effect of superoxide generation on resveratrol, the polyphenol was diluted to 5 mM in PBS containing 1 mM 4,4'-[azobis(oxymethylene]bis-benzoic acid (SOTS-1). In some experiments, resveratrol in PBS was supplemented with 1 to 5 units of tyrosinase (Sigma).

HPLC analysis of resveratrol

Resveratrol was analysed by HPLC using an LC-18 column (SUPELOCSIL, Sigma) and measuring absorbance of the eluent at 260 nm. 2 mM resveratrol was mixed in aqueous solution with or without the addition of 10 mM cysteine and then incubated at room temperature for 25 minutes in the presence or absence of UV. After incubation samples were immediately injected onto the HPLC column and analysed. The mobile phase consisted of a gradient of elution buffer containing 90% acetonitrile in H_2O and 0.1% trifluoroacetic acid, with a flow rate of 1 ml/min. For the initial 5 minutes of each run the mobile phase was kept constant at 50% elution buffer, which was then gradually elevated to 100% at 10 minutes, where it remained until at 20 minutes it was reduced to 0%.

NBT assay
To examine the redox cycling capability of resveratrol, the polyphenol at varying concentrations was added to 2 M potassium glycinate pH 10 in the presence or absence of 216 μM nitrotetrazolium blue chloride (NBT), with or without 10 units of superoxide dismutase. The superoxide released in the redox cycling of resveratrol reduced tetrazolium to its formazan form that was measured at 570 nm using a microplate reader.

Assessing protein modification by resveratrol
Cell lysates in 100 mM Tris-HCl pH 7.4, 0.5% Triton X-100 and protease inhibitors were centrifuged at 20,000 x g. Supernatants were then exposed to varying concentrations of resveratrol or resveratrol with tyrosinase. After 45 minutes incubation at room temperature each sample was supplemented with sample buffer containing 100 mM maleimide. Proteins in each sample were then resolved by SDS-PAGE and resveratrol modification visualised by exposing gels to UV irradiation (G:BOX Chemi XT Analyser).

Measuring BSA and GAPDH oxidation
Resveratrol at varying concentrations in PBS was supplemented with 100 μM bovine serum albumin (BSA) and/or 30 units of tyrosinase. In addition, samples containing only 100 μM BSA or 100 μM BSA with 30 units of tyrosinase were also generated. Samples were incubated for 20 minutes at room temperature and then analysed using monobromobimane (mBBr) or biotin-maleimide labelling. mBBr at 200 μM was added to each sample then incubated for 1 hour at 37°C before measuring its specific thiol-conjugated fluorescence (Ex394, Em490). Alternatively, sample were desalted (Zeba columns, ThermoFisher Scientific) and then 2 mM biotin-maleimide added for 1 hour at room temperature. After incubation, samples were supplemented with sample buffer containing 100 mM maleimide. In a separate experiment, GAPDH was reduced with 20 mM DTT for 2 min then diluted 450-fold before being treated with 100 μM resveratrol, 500 μM biotinylated glutathione (BioGEE) or combination of both. After 1 hour of incubation at room temperature, samples were supplemented with sample buffer containing 100 mM maleimide. These samples were resolved by SDS-PAGE and then probed using streptavidin-HRP.

Telemetric Blood Pressure Monitoring in Vivo.
Blood pressure (BP) was assessed by remote radiotelemetry in conscious freely moving mice. Briefly, mice were anesthetized with 2% isoflurane (Centaur Services) in 0.5 L of oxygen per minute with pre- and postoperative analgesia (buprenorphine, 0.1 mg per kg of body weight; Abbot Laboratories). A radiotelemetry probe catheter (TA11PA-C10, outside diameter of 0.4 mm; Data Science International) was implanted into the aortic arch of each mouse through the left carotid artery. The wellbeing of post-operative animals was monitored daily. After 10 days of recovery, mice housed individually in cages were placed above the telemetric receivers with an output to a computer. Blood pressure was recorded by scheduled sampling for 10 s every 5 min (Dataquest LabPRO Acquisition system version 3.01; Data Sciences International). Baseline measurements were collected for 4 consecutive days. A total of 5-6 WT or

KI mice per group were studied in two independent feeding studies to produce the average blood pressure data. Exclusion criteria included post-operative death (no animal excluded in the end of the study) and malfunction of telemetry device (3 mice in total were excluded due to the damage caused to the catheters).

Mass spectrometry analysis

10 mM cysteine or 1 mM resveratrol alone or in combination were incubated overnight at room temperature in 10 μM ammonium hydroxide (Sigma). After incubation, samples were analysed by electrospray ionization mass spectrometry in positive ion mode. To analyse modification of serum albumin, blood was taken from a vehicle or resveratrol fed mouse (~320mg/kg) and centrifuged to remove cells. The supernatant was then added to sample buffer and proteins resolved by SDS-PAGE. After staining proteins with colloidal Coomassie, the band corresponding to albumin was extracted for mass spectrometry analysis. A direct in-gel digestion with trypsin was performed on all excised gel bands, prior to subsequent analysis by mass spectrometry. Chromatographic separation was performed using an Ultimate 3000 NanoLC system (ThermoFisherScientific, UK). The eluate was ionised by electrospray ionisation using an Orbitrap-Fusion-Lumos (ThermoFisherScientific, UK) operating under Xcalibur v4.1. The MS/MS analyses were conducted using high-energy collision disassociation profiles that were chosen based on the mass-to-charge ratio (m/z) and the charge state of the peptide. This allowed us to identify the signature ions of resveratrol, m/z 91, 107, 119, 135 and 165 (+1) at the level of MS2, referenced from Thermo mzCloud database. Raw mass spectrometry data were processed into peak list files using Proteome Discoverer (PD v2.2; ThermoScientific). Processed data was then searched using Sequest search algorithm embedded in PD 2.2 against the current version of the reviewed SwissProt Mouse Albumin sequence downloaded from Uniprot (http://www.uniprot.org/uniprot/). All “PRM-tMS2” data were analysed and assigned manually within Xcalibur Qual Browser (ThermoScientific).

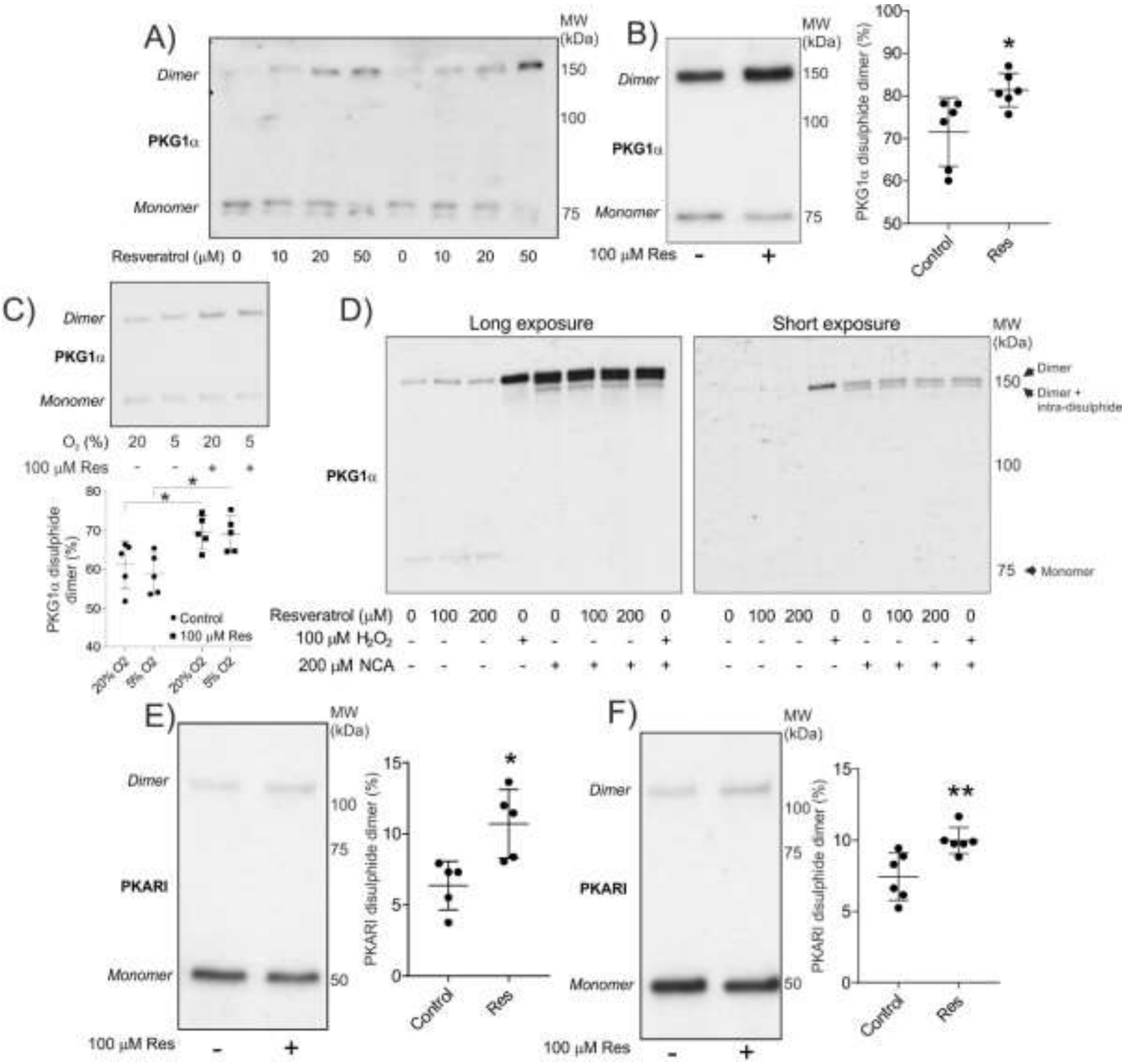

Supplementary Figure 1. Resveratrol induces protein oxidation. A) Resveratrol induces a concentration-dependent increase in PKG1α disulfide dimer in treated rat aortic SMC B) Treatment of human vascular SMC induces PKG1α disulfide dimerization (n=5-6). C) Resveratrol mediates PKG1α disulfide dimerization in cells cultured in 20% or 5% O_2 (n=5). D) Resveratrol mediates PKG1α disulfide dimer without intramolecular disulfide formation. Resveratrol also does not prevent intramolecular disulfide formation induced by 1-nitrosocyclohexyl acetate (NCA). Resveratrol induces PKARI oxidation in E) rat aortic SMC and F) human vascular SMC (n=5-6). * $p<0.05$, ** $p<0.01$. All data are presented as mean ± s.e.m. and analyzed using unpaired student's t-test (B, E, F) or using two-way ANOVA followed by Sidak post-hoc test (C)

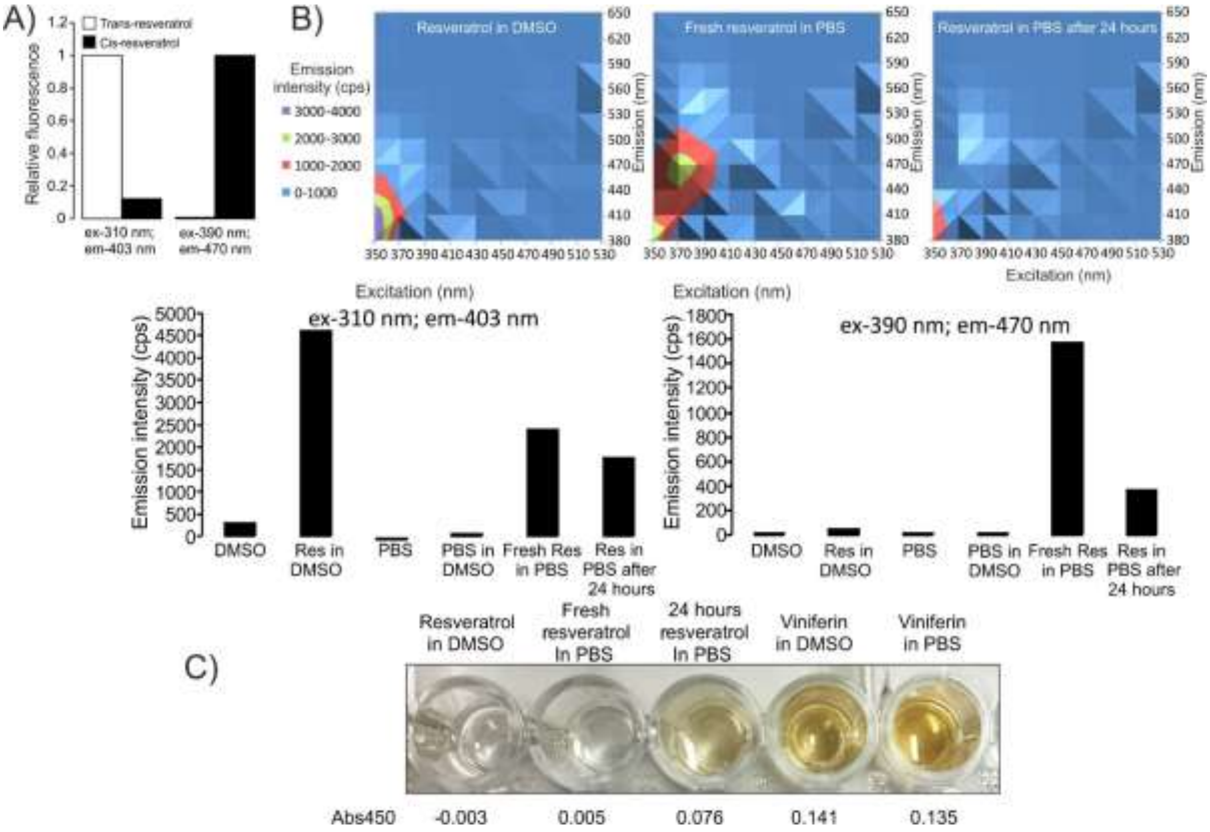

Supplementary Figure 2. Characterization of resveratrol. A) Fluorescent properties of commercial standards allows specific measurement of *trans* and *cis* resveratrol B) Heat map for excitation and emission spectre showing detection of *cis* form upon addition of resveratrol to an aqueous solution that is lost over 48 hours. C) Comparison of the 450 nm absorbance of resveratrol and its dimer viniferin.

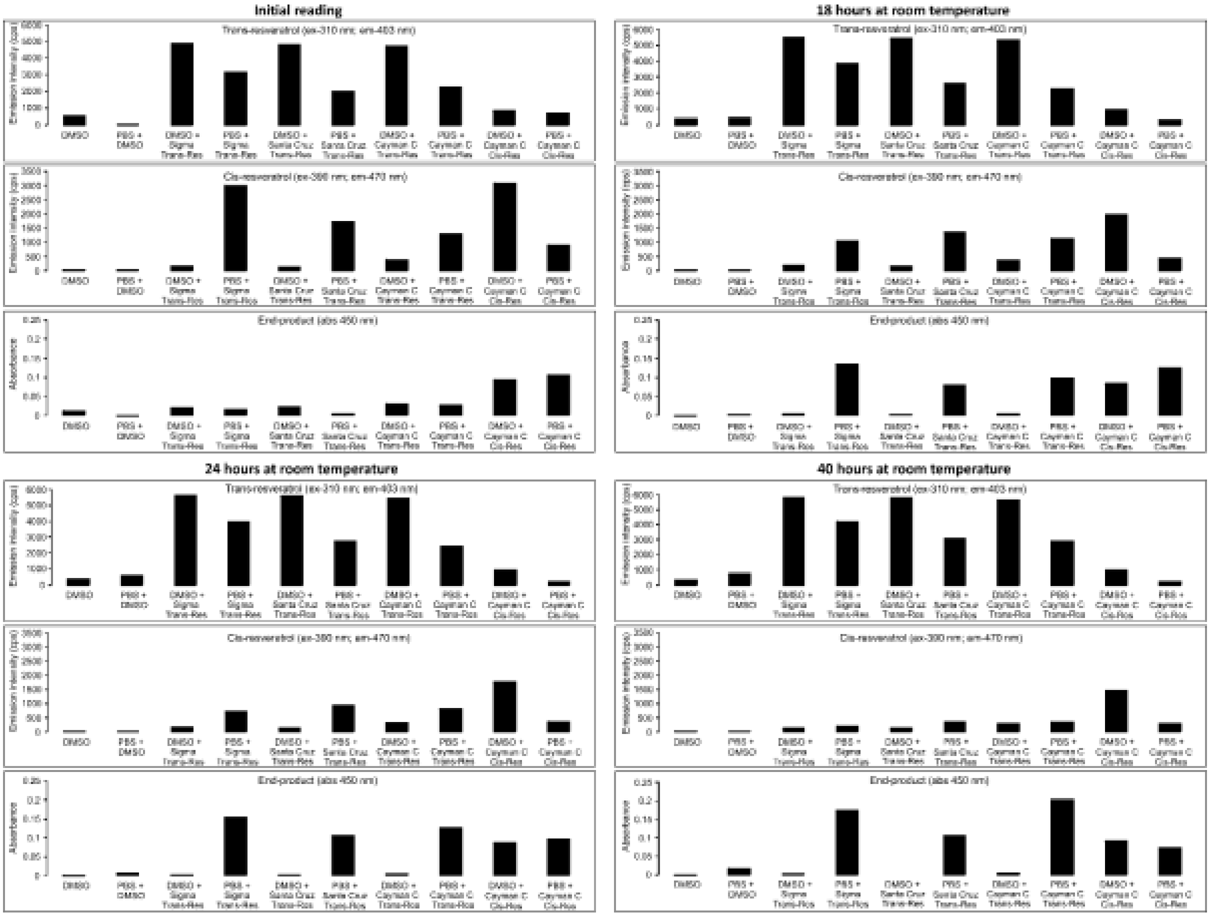

Supplementary Figure 3. Comprehensive evaluation of resveratrol. For each commercial source of resveratrol, the addition to an aqueous buffer causes immediate isomerization of the *trans* to the *cis* form. Over time this *cis* isomer is lost with accumulation of an end-product detected at 450 nm.

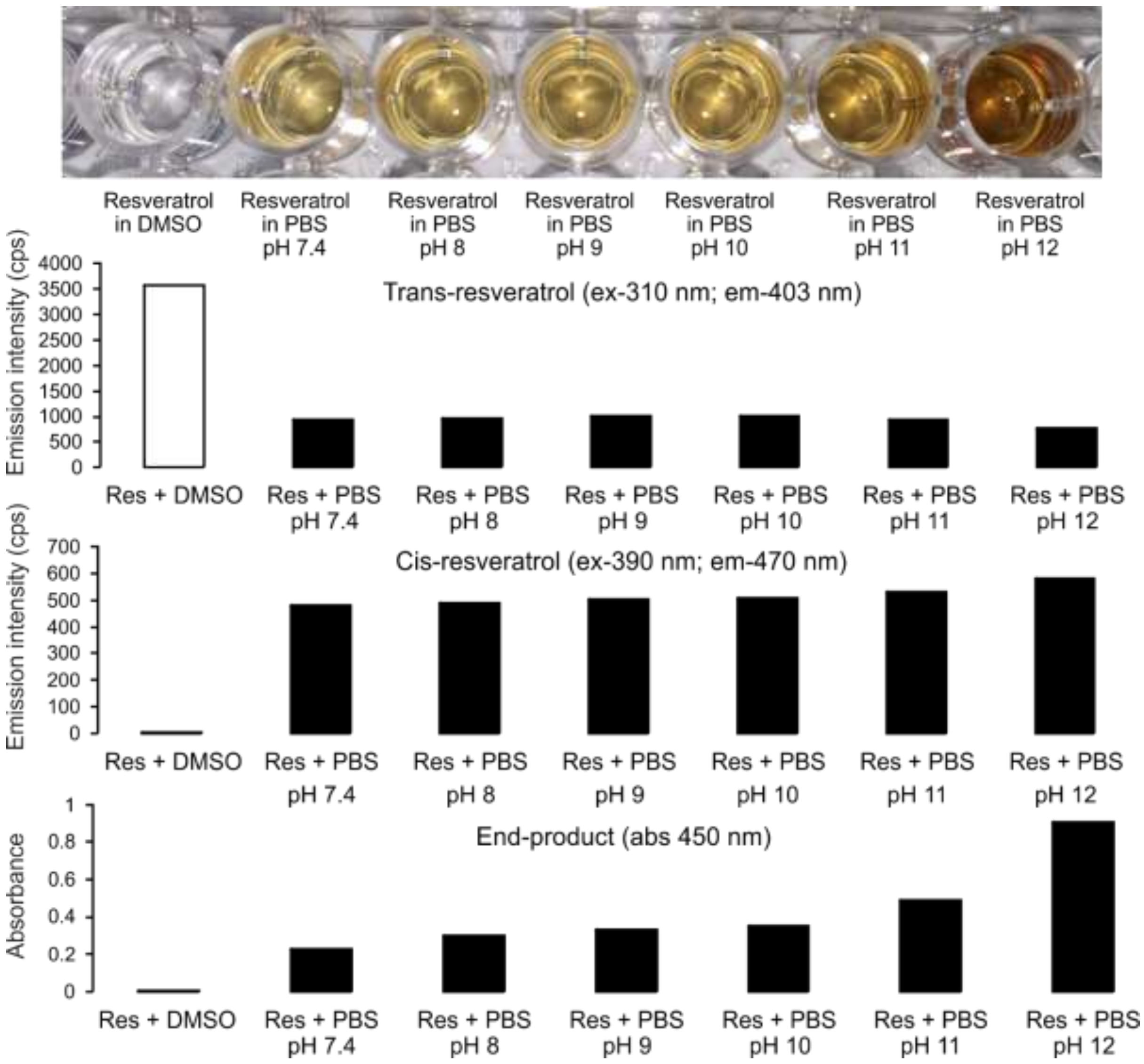

Supplementary Figure 4. Formation of the end-product of resveratrol is enhanced in a pH-dependent manner.

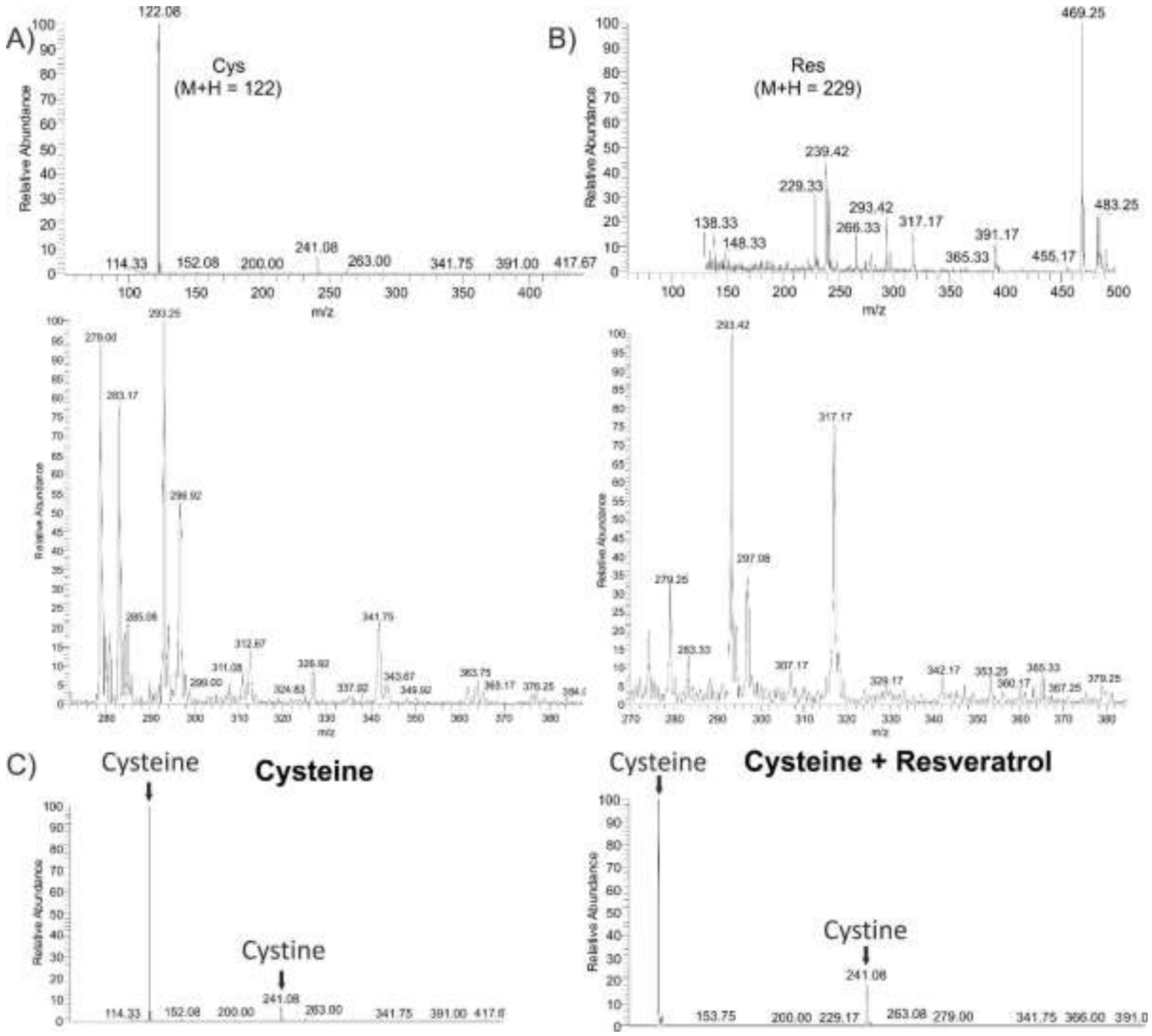

Supplementary Figure 5. Resveratrol adduction to cysteine thiols. A) Product spectra from positive-ion electrospray mass spectrometry analysis of cysteine alone. Products between 100 - 500 m/z (upper panel) and 270 - 390 m/z (lower panel). B) Product spectra from positive-ion electrospray mass spectrometry analysis of resveratrol alone. Products between 100 - 500 m/z (upper panel) and 270 - 390 m/z (lower panel). C) Product spectra from positive-ion electrospray mass spectrometry shows resveratrol increases the relative abundance of cystine.

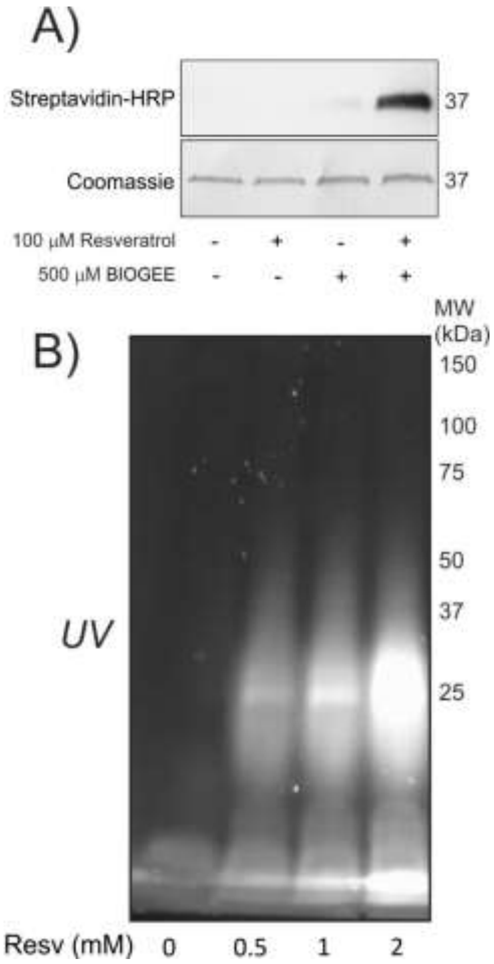

Supplementary Figure 6. Modification of proteins by resveratrol. A) Resveratrol enhances the glutathiolation of GAPDH by biotinylated glutathione (BIOGEE). B) Proteins modification by resveratrol increases in a concentration-dependent manner in cell lysates, observed after SDS-PAGE separation followed by exposure of proteins to UV irradiation.

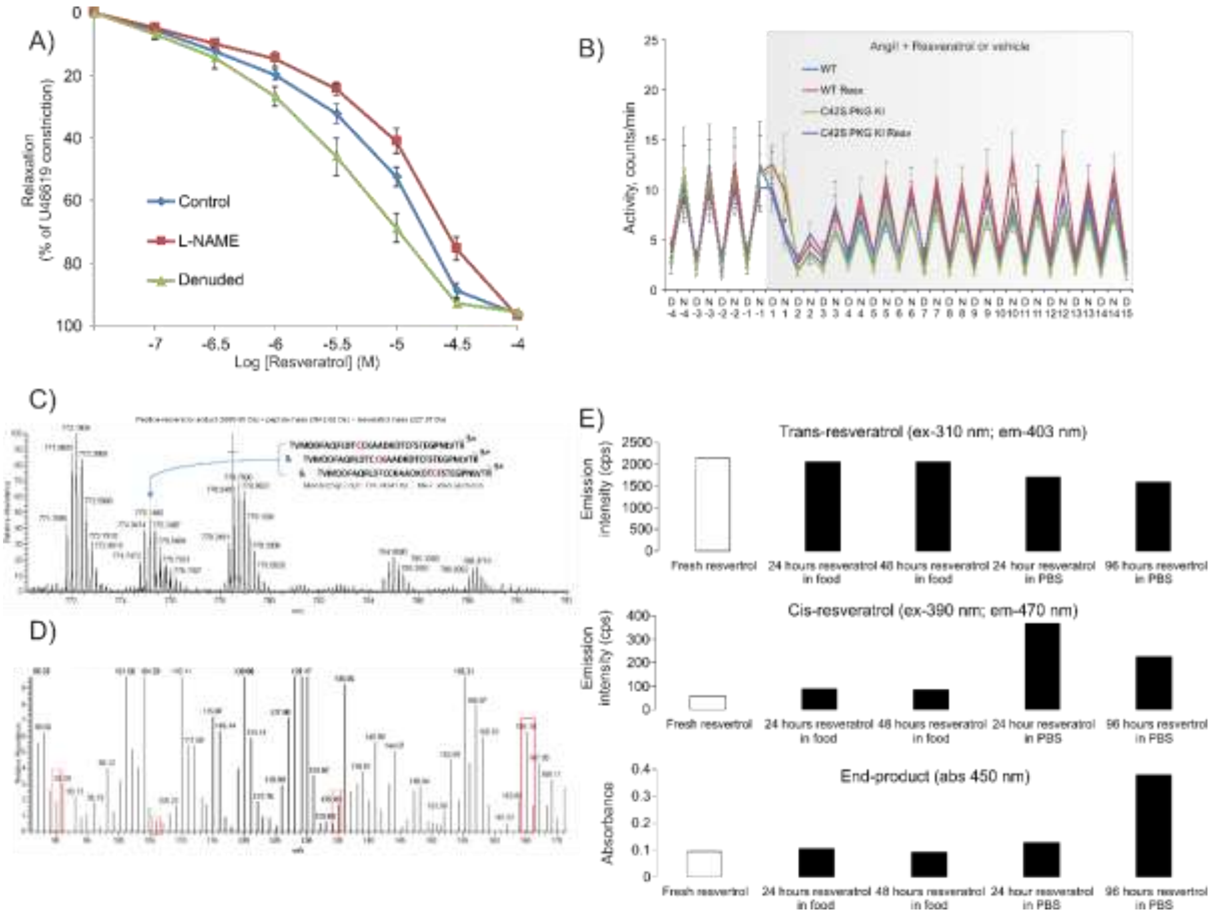

Supplementary Figure 7. Resveratrol feeding study. A) Resveratrol induces relaxation in denuded or L-NAME-treated vessels that is comparable to control tissue. B) Feeding of resveratrol does not impact on mouse activity. C) Product spectra for plasma albumin isolated from a mouse fed resveratrol with a peptide mass (774.74 Da) consistent with bound polyphenol. D) Fragmentation of the product ion from C) has signature (highlighted in red) that confirms the presence of resveratrol. E) Resveratrol remained largely stable in its *trans* form in food preparations over 48 hours.
